# Editorial: Clinical phytopharmacology

**DOI:** 10.3389/fphar.2023.1353483

**Published:** 2024-01-15

**Authors:** Merlin L. Willcox, Chi-Jung Tai, Kaushik Chattopadhyay, Xiao-Yang Hu, Michael Heinrich

**Affiliations:** ^1^ Primary Care Research Centre, Faculty of Medicine, University of Southampton, Southampton, United Kingdom; ^2^ Department of Family Medicine, School of Medicine, College of Medicine, Kaohsiung Medical University, Kaohsiung, Taiwan; ^3^ Lifespan and Population Health, School of Medicine, University of Nottingham, Nottingham, United Kingdom; ^4^ The Nottingham Centre for Evidence-Based Healthcare: A JBI Centre of Excellence, Nottingham, United Kingdom; ^5^ Pharmacognosy and Phytotherapy Group, UCL School of Pharmacy, London, United Kingdom; ^6^ Chinese Medicine Research Center, College of Chinese Medicine, China Medical University, Taichung, Taiwan

**Keywords:** clinical studies, phytopharmacology, traditional medicine, quality assurance, CONSORT, ConPhyMP

It is now outdated to state, as many medical doctors still do, that “there is no evidence for herbal medicines”. Since the new millennium, there has been a great increase in the number of clinical trials of medicinal plant preparations, and systematic reviews, some of which show promising results (Davidson et al., 2013; Timmer et al., 2013; Hu et al., 2017; Anheyer et al., 2018; Willcox et al., 2021b; Chattopadhyay et al., 2022). The Research Topic of papers in this Research Topic bears testimony to the many studies assessing medicinal plant preparations in a clinical context. While only five are formally included in the Research Topic, Frontiers in Pharmacology has published a large number of clinical studies and systematic reviews and meta-analyses.

However, progress has slowed down since the COVID-19 pandemic, as many trials were postponed. The increased use of remote consultations also made recruitment more difficult for trials which require face-to-face clinical assessment (Willcox et al., 2023). This might explain why this special Research Topic had relatively few submissions. We hope that this downturn has been temporary and that clinical trials of promising herbal medicines will again be prioritised.

High-quality randomised controlled clinical trials (RCTs) are essential for determining the effectiveness and safety of any treatment. They form the basis for systematic reviews and meta-analyses which inform evidence-based clinical guidelines for health workers (Chattopadhyay et al., 2023). Many studies on herbal medicine assess activity using *in-vitro* effects or animal models, but their results do not correlate very well with results from clinical trials (Willcox et al., 2011). *In vitro* studies may produce “false positive” results if the substances active *in vitro* are poorly bioavailable or are converted to inactive metabolites in humans. Conversely, they can produce “false negative” results if the active substances in humans are metabolites, and their parent compounds are inactive. Many animal models also produce misleading results as their metabolism and diseases differ significantly from those of humans. For example, penicillin is toxic to guinea pigs and may never have been deemed safe to use in humans, if it had first been tested in guinea pigs rather than mice (Stuart and Slavin, 1951). Many natural products which are very safe for human consumption, such as grapes, onions and chocolate, are highly toxic at small doses for cats and dogs (Cortinovis and Caloni, 2016). Furthermore, there are significant ethical concerns about animal experimentation. Currently, many studies cause unjustified suffering which does not result in any clinical benefit for human patients (Akhtar, 2015; Zhao et al., 2020) and the justification for such studies is often unclear.

Traditional herbal medicines have significant advantages over novel chemical compounds because they have been used in humans for hundreds or thousands of years. Important safety issues have already been identified through this long experience of use, so potentially toxic compounds and plant parts are already well known. Serious adverse reactions to traditional herbal preparations are extremely rare (Farah et al., 2000). Furthermore, indigenous knowledge and experience of traditional use is a very efficient way of identifying potentially effective treatments, which can result in new medicines being developed much faster and at a fraction of the cost of conventional pharmaceuticals, and which are more acceptable to local populations (Graz et al., 2005). Nevertheless, better methods are needed to prioritise herbal preparations for clinical trials, to maximise the chances of selecting effective preparations. Clinical trials are costly and time-consuming and it is simply impossible to conduct clinical trials on all traditional herbal preparations.

Retrospective Treatment-Outcome studies (RTOs) (Graz et al., 2005) can help to identify the herbal medicines associated with the best clinical outcomes (Xia et al., 2023); this method relies on patient-reported outcomes, so is only useful for conditions in which patients can identify whether the treatment is having an effect. Where patient records are computerised, Real-World Evidence (RWE) using a Real-World Database (RWD) of clinical use is another promising method for selecting potentially effective treatments for clinical trials. Computer databases in Traditional Chinese Medicine (TCM) hospitals record the formulation and dose of herbal products prescribed (Shao et al., 2021). This data can be linked to clinical information such as diagnosis and outcomes. However, designing a comprehensive study of a specific medicinal plant using a RWD is more challenging than pharmacoepidemiologic studies of pharmaceuticals. RWDs usually do not contain information on the chemical composition of the herbal products. Herbal extracts of the same plant part from different producers could contain different concentrations of the active components because of variations in agricultural and manufacturing processes (Tai et al., 2015). This will make it harder to find a clear dose-response relationship in data from RWDs. Adherence is another important Research Topic in evaluating the long-term effects of any medications. Therefore, although RWE studies can provide useful indications, these are not the most reliable evidence on effectiveness of herbal products.

Over the last 30 years with the development of the CONSORT guidelines on reporting clinical trials and their outcomes, we have clear guidelines on how to conduct and report clinical trials (http://www.consort-statement.org/ Consolidated Standards of Reporting Trials). These guidelines focus on transparency in reporting and the wider reproducibility of the research outcomes. However, robust RCTs on traditional herbal medicines are even more challenging to conduct. Pragmatic RCTs can be done on individualised treatments (Flower et al., 2019), but these can be difficult to scale up and results may not be easy to generalise beyond the practitioners involved in the study. In RCTs of a specific medicinal plant or herbal formula, high-quality standardised preparations are needed. In the EU and UK, the product needs to have EU-Good Manufacturing Practice (GMP) certification before a definitive trial can be approved. In the United States of America, an investigational drug application is needed which includes ascertaining GMP (https://www.fda.gov/drugs/types-applications/investigational-new-drug-ind-application). In the United Kingdom, this is not required for a feasibility trial, because it is not powered to produce statistically significant results to demonstrate effectiveness. However, a feasibility trial can be very useful to determine the acceptability of the herbal preparation in patient groups who are not accustomed to taking it, and the feasibility of delivering it in different ways. For example, what would conventional medical doctors in the United Kingdom feel about giving a herbal medicine, and how many of their patients would accept this instead of, or as well as, conventional treatment (Flower et al., 2019; Soilemezi et al., 2020; Willcox et al., 2021a; Willcox et al., 2023)? The feasibility trial also helps to identify important challenges in recruiting patients, follow-up, collecting and prioritising outcome measures, which can greatly improve the development of a definitive trial protocol (Willcox et al., 2021a; Willcox et al., 2023). However, moving from a feasibility trial to a definitive trial has also been challenging in recent years. As a result of the COVID-19 pandemic, sales of some products increased, so their manufacturers may feel they have little to gain from further clinical trials (Anon, 2022). Other promising herbal medicines are not currently available in any GMP preparations on the EU or United Kingdom market (Hu et al., 2017).

When a GMP preparation is available for a promising herbal medicine, which is not deemed to have sufficient evidence to justify a recommendation in clinical guidelines, the top priority must be to conduct a high-quality definitive RCT. Unfortunately, recent systematic reviews show that many clinical trials of herbal medicine are still of low quality and/or have poor reporting (Willcox et al., 2021b; Yan et al., 2021; Chattopadhyay et al., 2022; Zhang et al., 2022). Some problems could easily be avoided. There are specific guidelines for reporting trials of herbal medicines (Gagnier et al., 2006), which, however, lack a focus on reporting the composition of the material under investigation (Heinrich et al., 2022). Failure to report the composition in detail gives rise to safety and thus ethical concerns. Unfortunately, it seems that many clinical researchers are not aware of the need for detailed reporting of the herbal preparation under investigation. In case of polyherbal preparations, the situation is even more challenging. In future trials it is absolutely essential that the composition of the preparation used in the trial is reported in detail, including a phytochemical characterisation ideally quantifying some important active metabolites. Frontiers in Pharmacology has implemented the ConPhyMP guidelines which provide a framework for reporting the ‘phytochemical composition of medicinal plant preparations used in clinical, pharmacological and toxicological research’ (Heinrich et al., 2022). An open-access tool hosted by the Society for Medicinal Plant and Natural Product Research (GA) for checking manuscripts is available: https://ga-online.org/best-practice/. Failure to follow these guidelines was a major reason for manuscripts being rejected from this Research Topic.

Careful attention must be paid to Risk of Bias guidelines to ensure that risk of bias is as low as possible (Sterne et al., 2019). Most of these guidelines should not present a challenge for standardised herbal medicines. Participants can be assigned to trial arms through true randomisation (such as a computer-generated random number sequence) and allocation can be concealed at least until the participant has signed informed consent. Proper randomisation should ensure that baseline characteristics are comparable between groups at baseline (not only sociodemographic but also disease specific characteristics). Blinding is achievable where a matching placebo can be produced (especially for tablets and capsules) but can be challenging for herbal preparations with a distinctive taste. Close collaboration with the herbal manufacturer is essential for this (Flower et al., 2019). Nevertheless, many patients do not know what taste to expect, so inert colouring and flavouring can be enough to mimic a “herbal” preparation and produce effective blinding for both patients and researchers (Willcox et al., 2021a). Apart from the herbal medicine being tested, the groups should receive identical treatment and outcomes should be measured in the same way, using valid and reliable measures. Any deviations from the protocol should be recorded and reported. A primary outcome must be pre-specified and the minimum clinically important difference in this outcome between treatment and comparison groups must be used to calculate the sample size. Follow-up of participants must be as complete as possible, and any reason for differences in follow-up between groups must be explored. Statistical analysis should follow a pre-determined plan using robust methods, including intention-to-treat analysis of post-intervention differences in outcomes between trial arms. Comprehensive and transparent reporting of the trial methods, results and funding is required (Schulz et al., 2010). Again, this was a core reason for not accepting a manuscript.

The world is facing a massive increase in non-communicable diseases (Saeedi et al., 2019) and new waves of infections due to antimicrobial resistance (Murray et al., 2022), so it is more important than ever to redouble efforts to find more effective and affordable treatments both for acute illnesses and chronic conditions. Many patients prefer to take herbals rather than pharmaceuticals, so providing a choice of evidence-based treatments is likely to improve adherence (Sriraman et al., 2023). High-quality clinical trials of promising herbal medicines, which already have observational evidence of safety and effectiveness, could yield new treatments more quickly and less expensively than animal studies or the development of novel biomolecules.

Unfortunately, pharmaceutical companies often lack a compelling incentive to undertake clinical trials on traditional herbal medicines due to the inability to patent previously published knowledge (Chaudhary and Singh, 2012). Moreover, the presence of a conflict of interest poses another challenge, leading many to scrutinize the favourable outcomes of commercially funded clinical trials. Therefore, public funding is crucial for the conduct of high-quality clinical trials of herbal medicines. Such publicly funded research opportunities are expanding but remain notably constrained. Allocation of public research funding should be reviewed, to encourage scientists to exploit this important opportunity.

The papers in this Research Topic provide examples of how to improve the quality of design and reporting and, more generally are a call for a rigorous development of transdisciplinary research. Clinical research on phytopharmaceuticals requires collaboration not only at a clinical level, but also with traditional practitioners, experts in phytopharmaceutical analysis and plant scientists/pharmacognosists. A core ethical foundation of any clinical research is good evidence for safety. With the long tradition of use of many herbal medical preparations and if the quality of the material used is ascertained, clinical studies should focus on the most widely used and important medicinal plants and preparations and will provide a much better evidence base for their use.
